# Supplementary material for: Hierarchical on-surface synthesis and electronic structure of carbonyl-functionalized one- and two-dimensional covalent nanoarchitectures
Source: Nat Commun. 2017 Mar 21;8:14765. doi: 10.1038/ncomms14765 (PMC5364392; doi:10.1038/ncomms14765)
Supplement: Supplementary Information — Supplementary Figures, Supplementary Discussion, Supplementary Methods and Supplementary References. [file ncomms14765-s1.pdf]

Supplementary Discussion

Scanning tunneling microscopy data

Structural model of the CTPA macrocycles and chains

Supplementary Fig. 1a-c show STM images of the macrocycles and 1D chains. The blue boxes highlight the unit cell of each self-assembly as described in the main text. In addition, structural models of the macrocycle and chains are superimposed in Supplementary Fig. 1b, c. The center-to-center distances between the central N atoms measures 1.01 nm ± 0.03 nm for both the chains and the macrocycles, which is in good agreement with the center-to-center distance previously reported in dimethylmethylene-bridged triphenylamine 2D polymers on Ag(111).[1] The macrocycles and chains are arranged such that they are stabilized by C-H···O=C hydrogen bonds. In the case of the chains, the lateral displacement between neighboring chains differs: The C-H···O=C hydrogen bond length is different on each side of the CTPA unit. This is also confirmed in DFT (PBE+D3) optimized self-assembled CTPA chains in vacuum (Supplementary Fig. 1d). The unit cell size of the DFT (PBE+D3) optimized chains structures in given in Supplementary Fig. 1d, e.

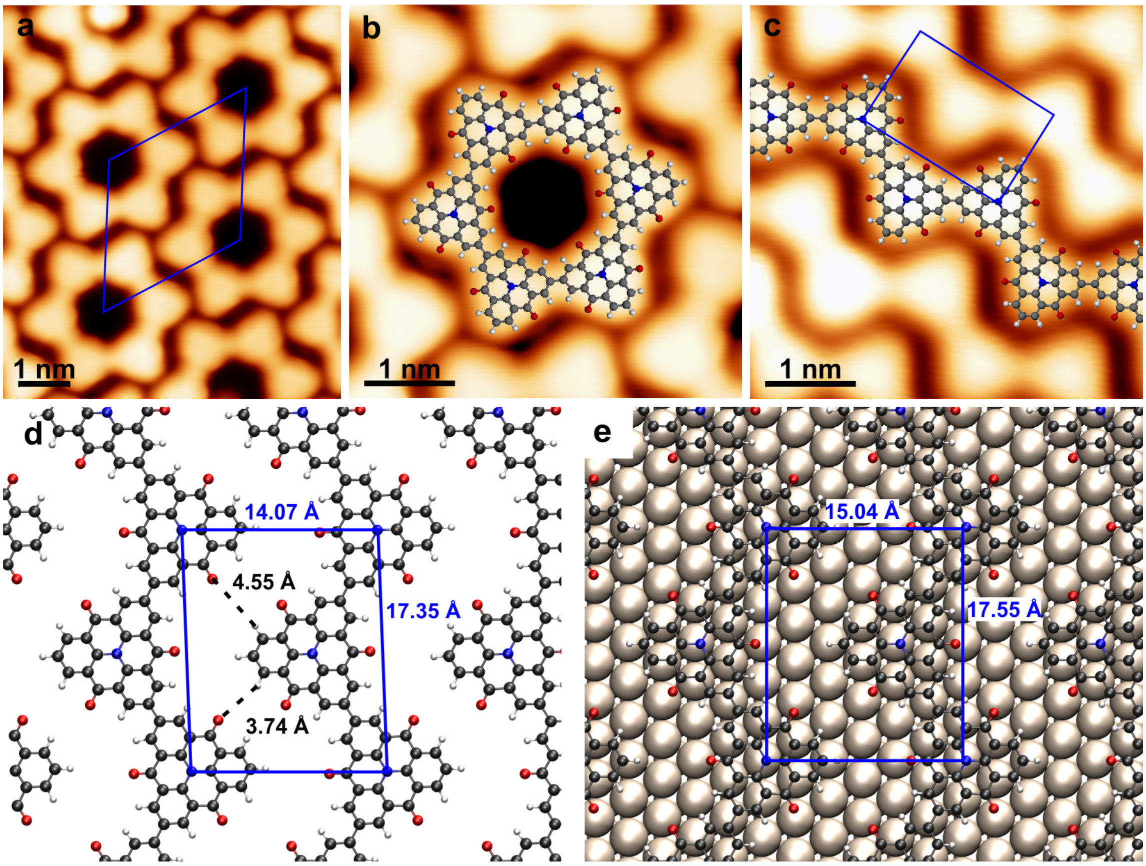

Supplementary Figure 1 | Structural model of the CTPA macrocycles and chains. (**a**) Self-assembled CTPA macrocycles on Au(111). The unit cell is highlighted in blue. (**b**)-(**c**) Structural model of the covalently bonded macrocycles and chains, respectively, is superimposed on corresponding STM measurements. The blue box depicts a possible unit cell. (**d**)-(**e**) DFT (PBE+D3) optimized self-assembled CTPA chains in vacuum and on the Au(111) substrate. In the latter case, lattice dimensions are determined by the optimized Au(111) slab. STM parameters: (**a**, **b**) *I* = 420 pA, *V* = -160 mV (**c**) *I* = 100 pA, *V* = 100 mV.

High-resolution STM topographies of CTPA macrocycles and chains

Submolecular imaging in SPM[2-5] is a convenient tool to analyze the chemical structure of molecules. In order to resolve the submolecular structure of the polymers, we used functionalized tips. While mostly CO-functionalized tips are used in non-contact atomic force measurements,[6, 7] we applied voltage pulses on co-adsorbed amino-substituted carbonyl-bridged triphenylamines. Supplementary Fig. 2 shows STM images (original and inverted low-pass / Laplace filtered) of the macrocycles and chains recorded with such a functionalized tip. Features related to the phenyl rings of the CTPA backbone are revealed in the high-resolution images. The connection between the CTPA units within the chains and macrocycles appears as a covalent bond. The intramolecular resolution allows for an accurate measurement of the next-neighbor distances of 1.01 ± 0.03 nm.

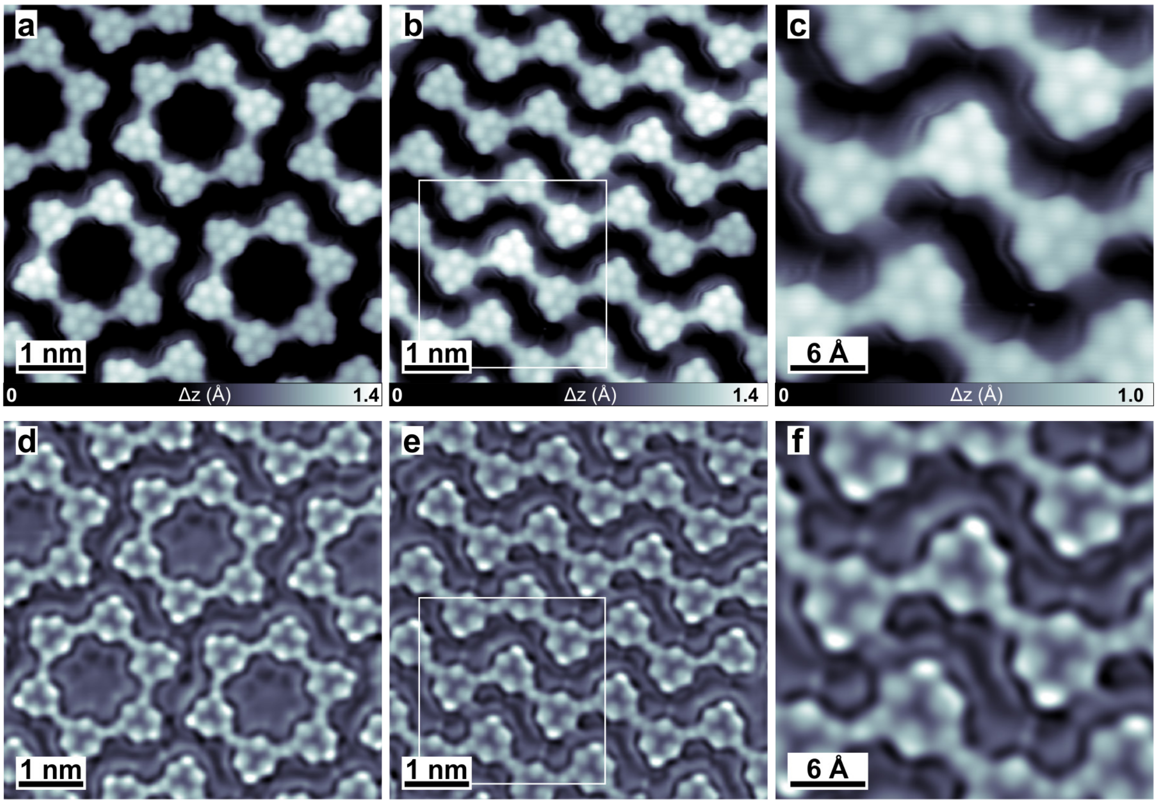

Supplementary Figure 2 | STM images of CTPA macrocycles and chains revealing submolecular features. High-resolution STM topographies of the macrocycles (**a**) and chains (**b**,**c**) recorded with a functionalized tip. (**d**)-(**f**) show the corresponding inverted low-pass / Laplace filtered images. STM parameters: T = 77.8 K, I = 1.3 nA, V = -50 mV.

Defect formation in the 2D CTPA polymer

Supplementary Fig. 3 shows examples of defects which might occur in the hierarchical on-surface synthesis of honeycomb networks using chains and macrocycles as intermediates. In the covalent linkage of 6-membered macrocycles, the only conceivable defect would be a highly strained pair of 4-8-membered macrocycles. These defects would lead to a C-C bonding angle of around 90°. However, both the high rigidity of the CTPA scaffold and the resulting macrocycles cannot compensate for that strain.
In contrast, short chains, such as dimers or trimers, can connect to chains and macrocycles and form defects. These defects, however, are only observed at the periphery of the honeycomb network. The amount of short chains (especially dimers and trimers) as intermediates determine the number of defects, and can be minimized by optimizing the growth parameters.

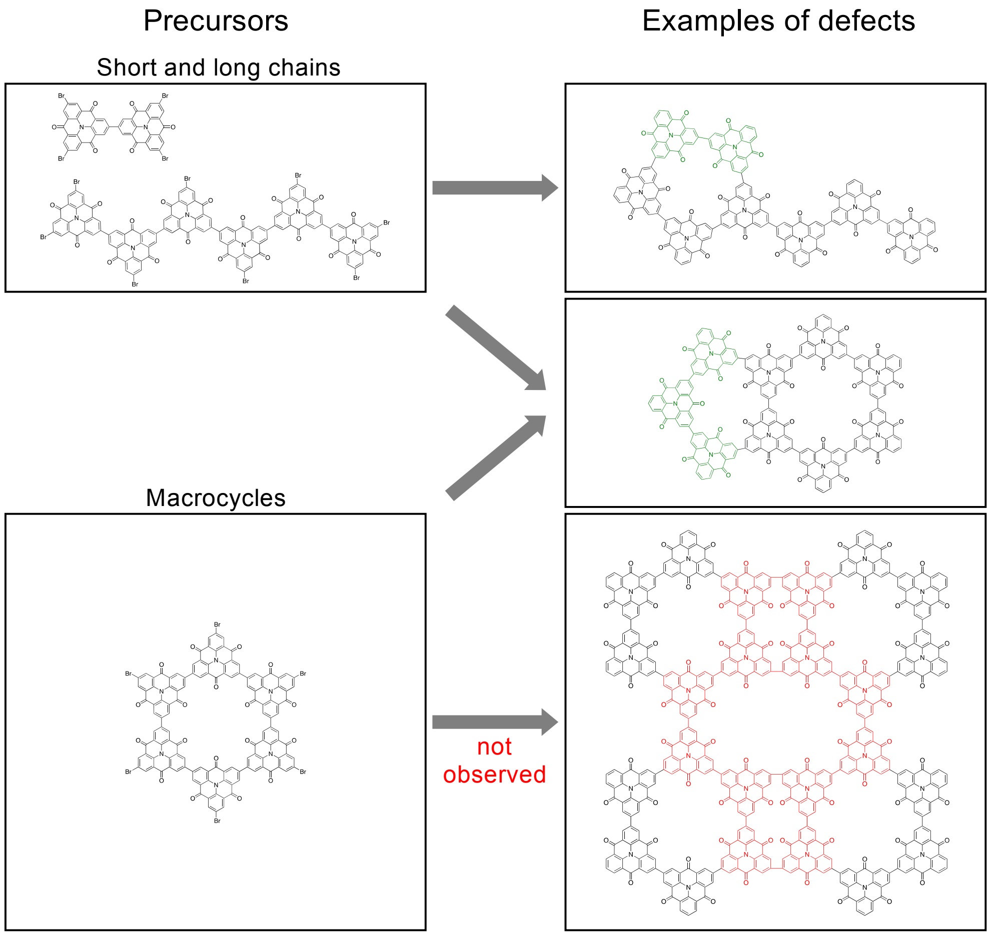

Supplementary Figure 3 | Defect formation in the 2D CTPA polymer. Scheme illustrating the formation of possible defects at the periphery of two-dimensional CTPA polymers during the second reaction step of the hierarchical synthesis.

STS – Distance dependence

In STS, the peak energies of the frontier orbitals can shift with changing tip-sample separation. In our experiment, the peak energies measured in STS were found to be independent of the tip-molecule separation, as seen from the STS spectra of the conduction band (CB) on the covalently-linked chains shown in Supplementary Fig. 4. The CB peak energy is at 1.66 ± 0.03 eV (set point: 700 pA, -3 V and *z*-offset: 0 pm). Upon increasing the *z*-offset with open feedback loop, the peak energy does not change more than ± 30 meV, indicating that there was no observable effect of the electric field in the tunnel junction.

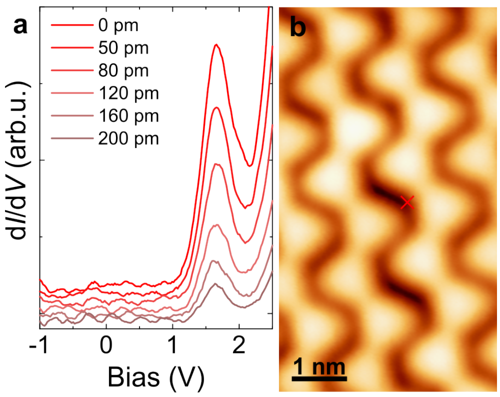

Supplementary Figure 4 | d*I*/d*V* spectra of CTPA chains at varied tip-sample distances. (**a**) Distance dependence of STS spectra on chains formed from CTPA **1** shown in (**b**). The peak position is not changed at different tip-sample separations. Open feedback parameters: (**a**) I = 700 pA V = -3 V, V_{mod} = 5 mV_{rms}, STM parameters: (**b**) V = 100 mV, I = 100 pA.

STS measurements on the CTPA monomer, 1D and 2D polymers

In this section, we compare the d*I*/d*V* spectra recorded at different positions along the CTPA polymers and the monomer **1**. In Supplementary Fig. 5a, we show STS spectra measured on the same structures as those discussed in the main manuscript between -2.5 and +3.0 eV. The spectra were measured on Au(111) (black), at the center of the molecule (red), on the newly formed C-C bond (blue) and aside of the C-C bond (green) (see corresponding topographies in Supplementary Fig. 5b). While at the center of the molecule the contribution of the CB states is weak, two distinct peaks are well visible in d*I*/d*V* measurements on the C-C bond and aside of the C-C bond. The peaks at 2.22 ± 0.03 V, 2.21 ±0.06 V, and 2.42 ±0.03 V, for 2D network, self-assembled chains and free-standing chain, respectively, measured on the C-C bond (blue) can be attributed to CB+2, and the peaks at 1.51 ± 0.03 V, 1.50 ±0.06 V, and 1.67 ± 0.03 V, for 2D network, self-assembled chains and free-standing chain, respectively, measured aside of the C-C bond (green) to the CB. For the monomer **1** the d*I*/d*V* spectra were measured at the center (red) and at the edge (green) of the molecule featuring two distinct peaks above the Au(111) reference spectrum (black) at -1.96 ± 0.03 eV and at 1.95 ± 0.03 eV. The peaks can be attributed to the highest occupied orbital (HOMO) and the lowest unoccupied orbital (LUMO) of the monomer, resulting in a HOMO-LUMO gap of 3.91 ± 0.03 eV.

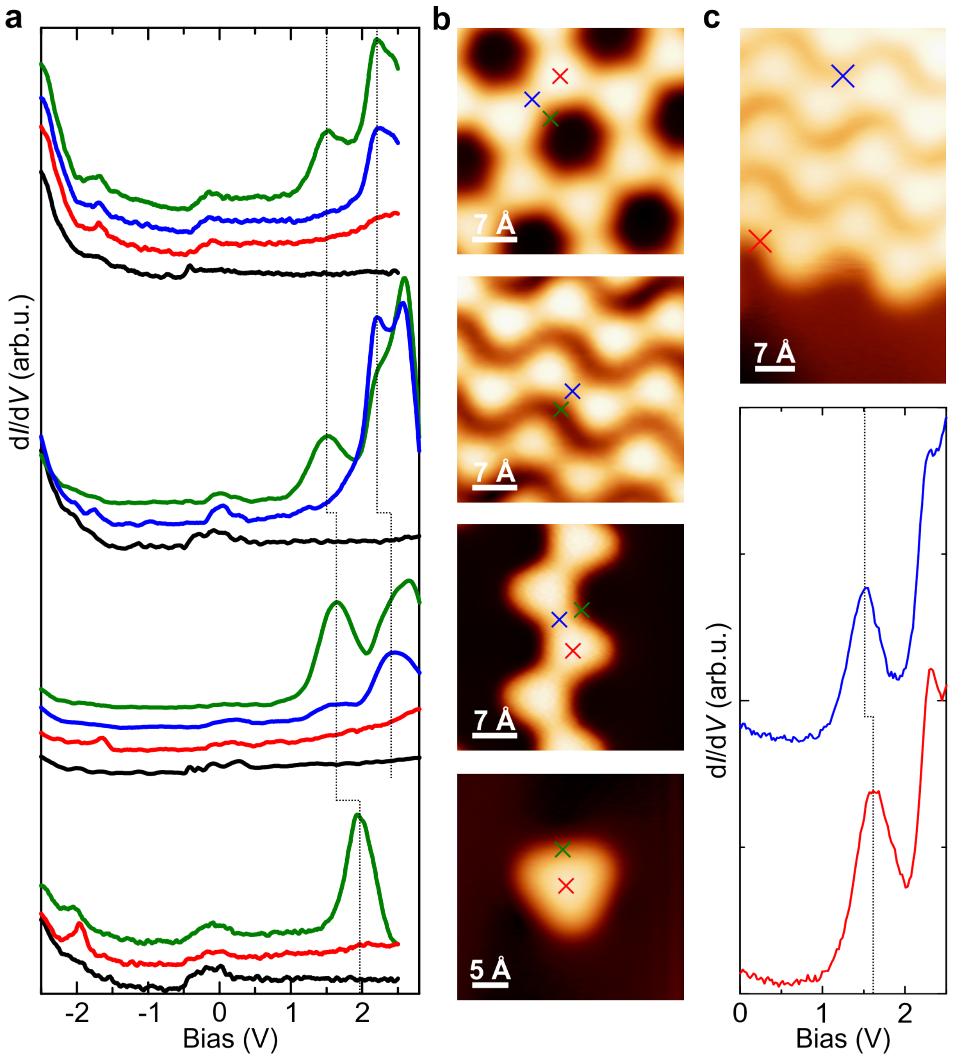

Supplementary Figure 5 | d*I*/d*V* spectra on the CTPA monomer, 1D and 2D polymers. (**a**) Shows the STS characteristics recorded on the polymers and the monomer **1** between -2.5 V and +3.0 V. The spectra were measured on Au(111) (black), at the center of the molecule (red), at the C-C bond (blue), and aside of the C-C bond (green, polymer) or at the edge of the monomer (green, monomer), as indicated in the STM topographies in (**b**). (**c**) STS measurements in the center and at the edge of a self-assembled island of CTPA chains. Open feedback parameters: V_{mod} = 12 mV_{rms}, (**a**, 2D) I = 700 pA, V = -4 V (**a**, 1D self-assembly, 1D and monomer) I = 50 pA, V = 1 V, (**c**) I = 100 pA V = 1 V. STM parameters: (**b**, 2D) I = 50 pA, V = -50 mV, (**b**, 1D self-assembly) I = 10 pA, V = 1 V, (**b**, 1D) I = 50 pA, V = 1 V (**b**, monomer) I = 50 pA, V = -500 mV.

In Supplementary Fig. 5c, we compare STS measurements at the edge (red curve) and within the self-assembly (blue curve) of 1D chains. At the edge of the self-assembly, the peak of the CB measures 1.61 ± 0.03 eV compared to 1.50 ± 0.06 eV within the self-assembly and hence shifts away from the Fermi level, towards the value measured for free-standing chains (1.67 ± 0.03 eV). This can be explained by the reduced number of H-bonds at the periphery of the self-assembly.

The Shockley surface state is measured both, above the bare Au(111) and the CTPA unit of the 2D network (Supplementary Fig. 6). We observe a shift towards the Fermi level of the surface state measured on the molecule compared to the Au(111) substrate of approximately 130 - 250 meV.

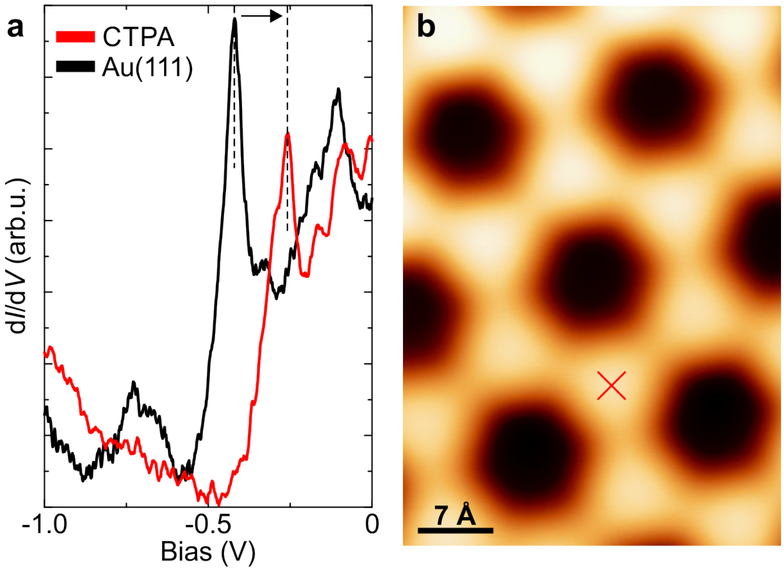

Supplementary Figure 6 | STS spectra of the Shockley surface state. (**a**) Comparison between the Shockley surface state measured on bare Au(111) (black) and on the CTPA network (red) with the corresponding STM image shown in (**b**). The surface state measured above the CTPA network is shifted towards the Fermi level. Open feedback parameters: (**a**) I_{set} = 240 pA, V = -0.4 V, V_{mod} = 6 mV_{rms}. STM parameters: (**b**) I = 50 pA, V = -50 mV.

Electron confinement in the porous molecular network

The surface state of the Au(111) surface can be confined using porous molecular architectures,[8, 9] which can be observed in an increased amplitude of the dI/dV signal in the pores compared to the bare substrate. In Supplementary Fig. 7 we measured STM topography and simultaneously recorded the dI/dV map with closed feedback near the surface state energy. The dI/dV signal is higher in the pores and at the rim of the polymer compared to the rest of the substrate. Therefore, we conclude that the surface state is weakly confined in the pores.

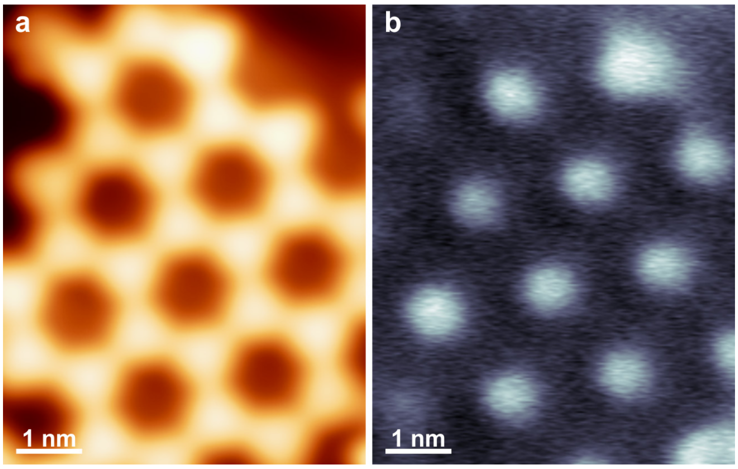

Supplementary Figure 7 | d*I*/d*V* map of the 2D CTPA polymer near the Shockley surface state. Surface state measured in the pores of the CTPA polymer: (**a**) Topography of the 2D CTPA polymer and (**b**) simultaneously recorded constant current dI/dV map at -500 mV. STM parameters: I = 150 pA, V = -500 mV, V_{mod} = 15 mV_{rms}.

Constant current dI/dV maps on the CTPA network, chain, and monomer

Here, we compare dI/dV maps in constant current (CC) and constant height (CH) mode for the CTPA polymers and the monomer **1**. On the 1D and 2D polymer, the differential conductance around the valence band edge (between -1.7 eV and -1.5 eV) in CC dI/dV maps is visible throughout the carbon backbone (Supplementary Fig. 8b, i), and is maximized in the center of the CTPA units. The dI/dV maps recorded at the conduction band energies, at 1.5 eV (CB, Supplementary Fig. 8c) and 2.2 eV (CB+2, Supplementary Fig. 8d) for the 2D, and at 1.7 eV (Supplementary Fig. 8j) and 2.4 eV (Supplementary Fig. 8k) for the 1D polymer, show pronounced features at the edge of the polymers and at the newly formed C-C bond, respectively. CC and CH dI/dV maps show qualitatively similar structures.

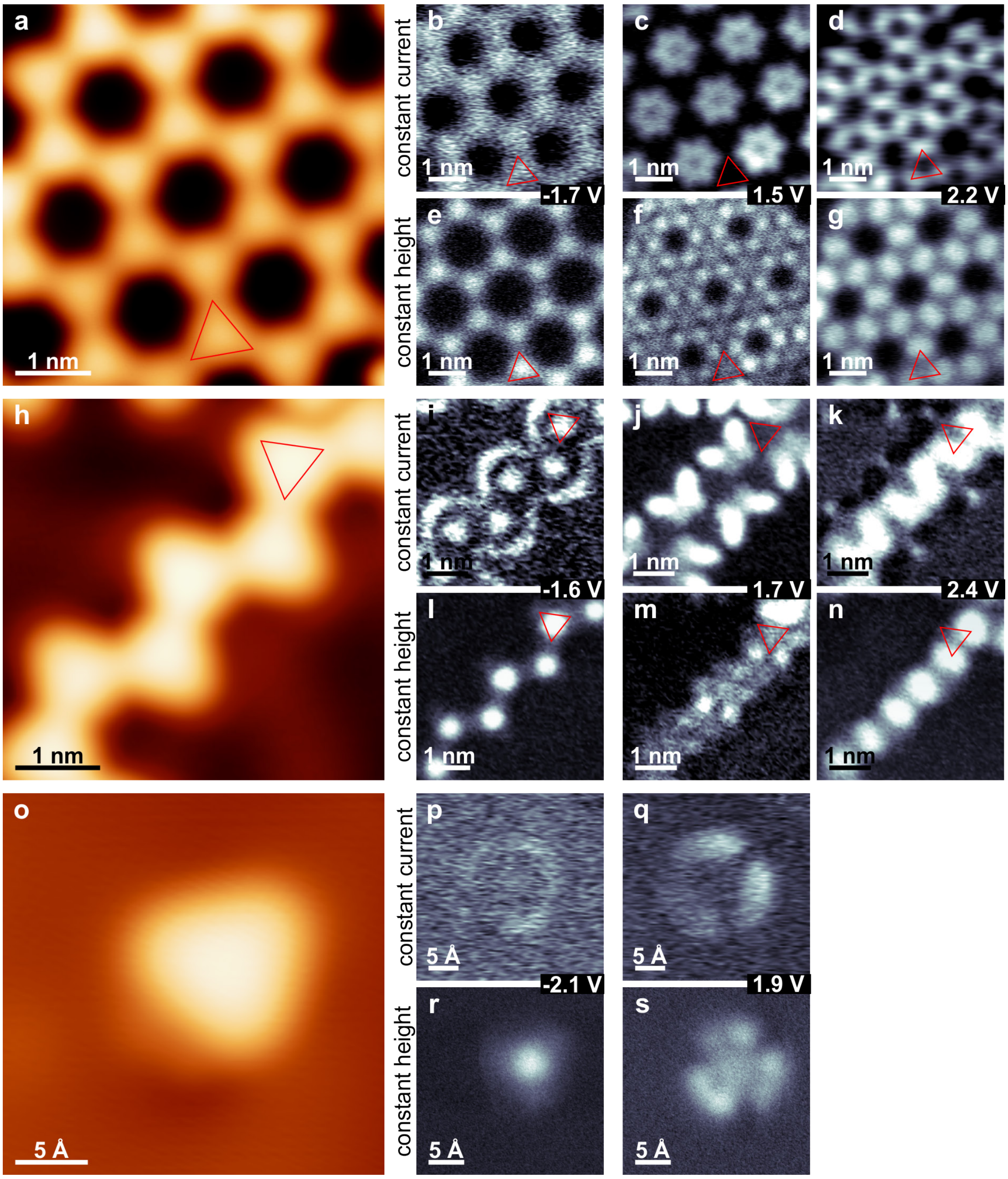

Supplementary Figure 8 | Constant current vs. constant height dI/dV maps of the CTPA monomer, chain, and polymer. Constant current (**b-d**) and constant height (**e-g**) dI/dV maps shown for the 2D polymer with the corresponding topography in (**a**), constant current (**i-k**) and constant height (**l-n**) dI/dV maps for the 1D polymer with the corresponding topography shown in (**h**), and constant current (**p**, **q**) and constant height dI/dV maps (**r**, **s**) for the monomer shown in (**o**). Both constant current and constant height maps reproduce qualitatively the same features. STM parameters: Open feedback parameters: I_{set} = 180 pA, V_{mod} = 12 mV$_{rms}$, closed feedback parameters: (**a**) I = 50 pA, V = -50 mV (**b-d**) I = 150 pA, V_{mod} = 15 mV$_{rms}$, (**h**) I = 180 pA, V = 100 mV, (**i-k**) I = 180 pA, V_{mod} = 12 mV$_{rms}$, (**o**) I = 180 pA, V = -500 mV, (**p**, **q**) I = 180 pA, V_{mod} = 12 mV$_{rms}$.

The CC dI/dV map of the monomer at the LUMO energy provided in Supplementary Fig. 8q shows the same features aside of the molecular scaffold as it is the case for the 1D chains (Supplementary Fig. 8j) and 2D networks (Supplementary Fig. 8c) at the CB. The contrast at the HOMO energy is less pronounced and is distributed homogeneously across the CTPA scaffold (Supplementary Fig. 8p) similar to the contrast of the 2D network (Supplementary Fig. 8b). However, the main features in CC maps of the 2D, 1D, and 0D structures are qualitatively similar to those measured in CH maps (Supplementary Fig. 8e-g, l-n, and r-s) that were discussed in the main manuscript. This observation is in accordance with the planar adsorption geometry of the 2D and 1D polymer on the surface.

Bias dependence of STM topographies

In Supplementary Fig. 9 we show the bias dependence of the STM contrast in constant current measurements of the 2D (Supplementary Fig. 9a-d), 1D (Supplementary Fig. 9e-h) polymer, and 0D monomer (Supplementary Fig. 9i-k). For negative biases below the VBE, the STM topography is dominated by the whole carbon backbone of the covalently-linked structures, which reflects the spatial distribution of the electronic states of the VB at the center of the CTPA units. In case of positive biases, the STM contrast is dominated by contributions of the CBs at the periphery of the C-C bond for bias voltages above the CBE and at the C-C bond for voltages above the CB+1. A similar contrast is observed for the monomer, where the STM topography contrast is changed at the periphery of the molecular scaffold for bias voltages close to the LUMO energy. The spatial distribution of the electronic states measured in differential conductance maps is well reproduced in the bias dependent imaging.

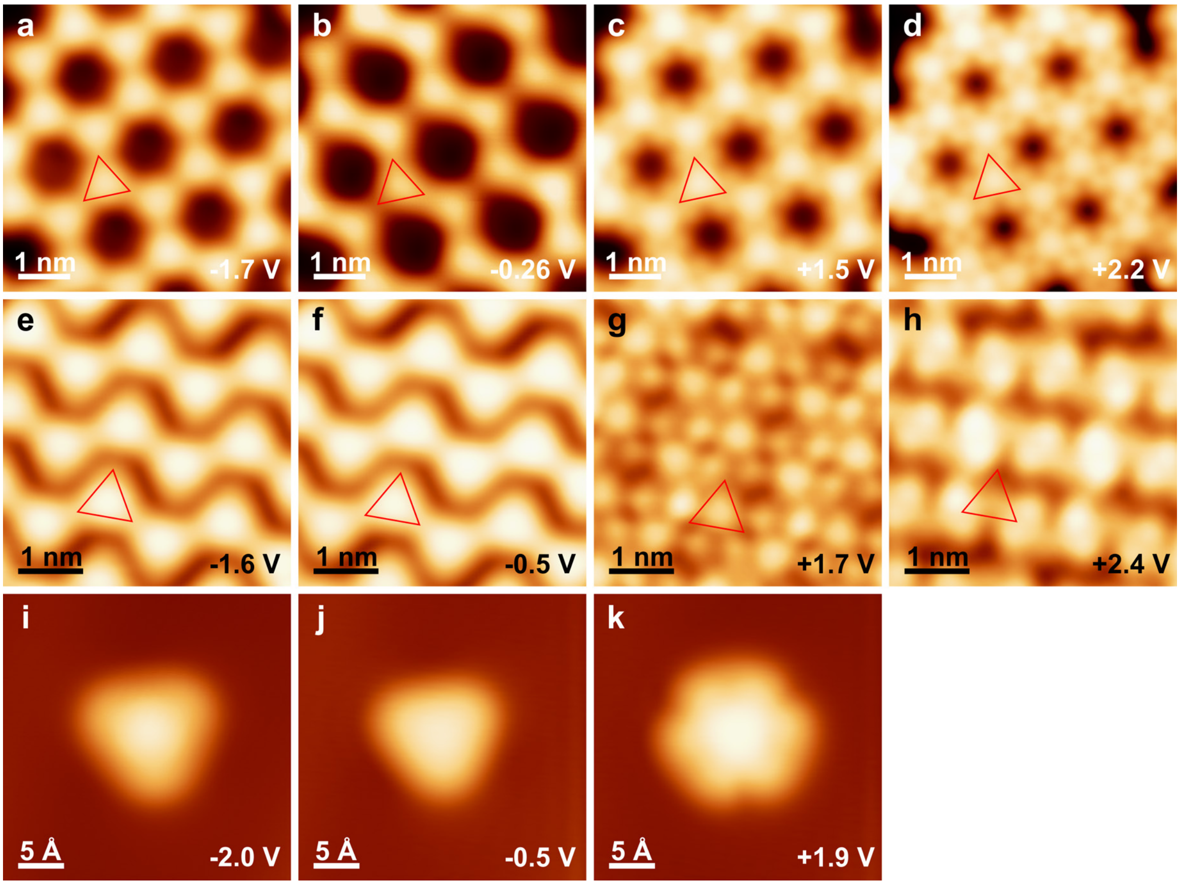

Supplementary Figure 9 | Bias dependent STM images of the CTPA monomer, chains, and polymer. Bias dependence of the STM topography on the 2D polymer (**a**-**d**), 1D self-assembled chains (**e**-**h**), and the monomer (**i**-**k**). The red triangles highlight the position of one CTPA unit. STM parameters: I = 180 pA.

DFT results

PBE calculated band structure

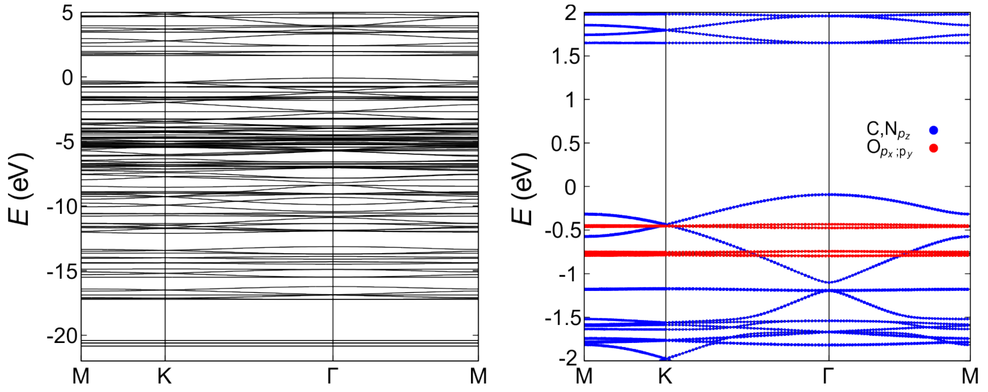

Supplementary Figure 10 | PBE+D3 calculated band structure of the free-standing 2D CTPA polymer. DFT calculated band structure of the free-standing 2D CTPA polymer showing mostly dispersion-less (flat) bands. For comparison, the close up around the Fermi level (Fig. 6e in the main text) is shown again.

HSE calculated band structures

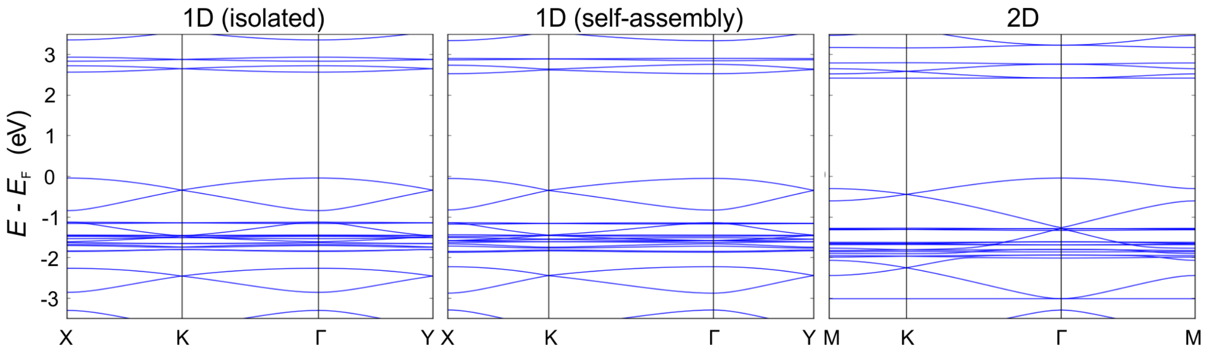

Supplementary Figure 11 | HSE calculated band structures of the free-standing CTPA polymers with different dimensionalities. The band gap is slightly decreased with increasing interactions in the two spatial directions. Besides that the band structures are similar. In particular, the changes between PBE and HSE results that are observed for the 2D case, e.g., the lower lying flat oxygen bands (compare Supplementary Fig. 10), are also present in the two 1D structures.

Charge density difference of the CTPA polymer on Au(111)

Supplementary Figure 12 shows the charge rearrangements due to Pauli repulsion caused by the pillow-effect[10] that is observed whenever an adsorbate approaches a surface. The magnitude of this effect is in very good agreement with what is found for physisorbed graphene on Au(111), whereas this effect is an order of magnitude larger for adsorbates at smaller adsorption distances, such as graphene chemisorbed on Ni(111).[11]

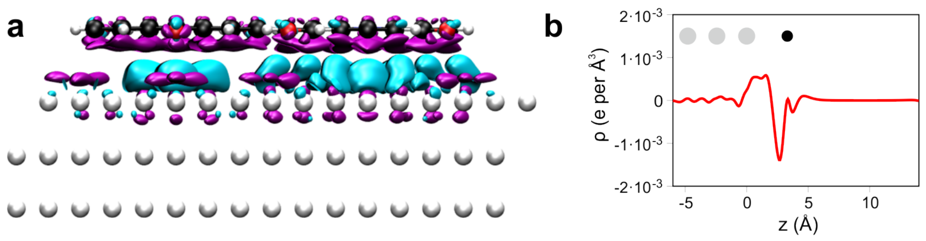

Supplementary Figure 12 | Charge density difference (CDD) of the CTPA polymer on Au(111). (**a**) 3D depiction of charge accumulation (cyan) and depletion (magenta) for an isodensity value of 0.003 e/Å^3. (**b**) 2D profile of the CDD along z averaged over xy planes. The position of the topmost surface atoms is chosen for z = 0 Å. Gray and black circles depict the z positions of surface layers and the CTPA polymer, respectively.

Band structure and partial charge densities of 1D and 2D polymeric CTPA structures in gas phase

The experimentally obtained decrease of the band gap upon increasing the dimensionality towards the 2D networks is nicely reproduced at the PBE+D3 level, with a calculated band gap of 1.84 eV for the 1D chains compared to a band gap of 1.74 eV in the 2D network. The effect of the reduced dimensionality is best visualized by comparing the band structures (Fig. 6b and e) of the two systems. First, due to the reduced symmetry, the high symmetry points at the sides of the Brillouin zone (M in Fig. 6e) are no longer symmetry equivalent and split into two sets of points X and Y in Fig. 6b (denoted according to their orientation in real space). In order to capture the dispersion visible for the 2D network, one, therefore, has to choose specific directions in reciprocal space, namely the one into which the linear chain is oriented, whereas the alternative paths along $\overline{\Gamma X}$ and $\overline{YK}$ carry dispersion-less bands. These bands are missing in the band structure of the 1D chain compared to the 2D case, which leads to an increased band dispersion between $\overline{XK}$ and a decreased band width along $\overline{KY}$ in the conduction and the valence band region. As a result, the VB at X is energetically closer, but the VBE remains at the Γ point also for the 1D structure. Analogous trends are also observed for the first conduction bands. This leads overall to a decrease of the band gap by 0.1 eV, which is in good agreement with the experimentally observed decrease of approximately 170 meV. These changes are also visible in the DoS (Fig. 6a and d), showing a decreased broadening of the VB and a decreased intensity of the CBE due to the missing, dispersion-less band. In addition to the described changes in the C,N_{pz} dominated bands, an increased splitting of the dispersion-less $O_{px,py}$ bands is observed.

In Supplementary Fig. 13, we provide partial charge densities for the 2D and 1D CTPA polymers in gas phase, analogously to the ones shown in Fig. 7d for the 2D structure on Au(111). Here, the densities are averaged over all **k** points throughout the Brillouin zone. The perfect agreement between Supplementary Fig. 13 and Fig. 7d shows, on the one hand, that such an averaging over **k** points, which is not easily possible on the surface, is not necessary to explain the experimentally observed features, and, on the other hand, demonstrates again the free-standing character of the electronic structure of CTPA polymer on Au(111). In both cases, the features reproduce the experimental STS maps recorded around the Fermi level for VB, CB, and CB+2 (see Fig. 5 in the main text). On closer inspection, the pairwise degeneracy of the unoccupied bands (CB, CB+1, and CB+2) is broken in the 1D case, showing some levels at shifted energies. The superposition (not shown) of the bands of the 1D case that are degenerate in the 2D case, however, reproduces the partial charges density of the latter case perfectly.

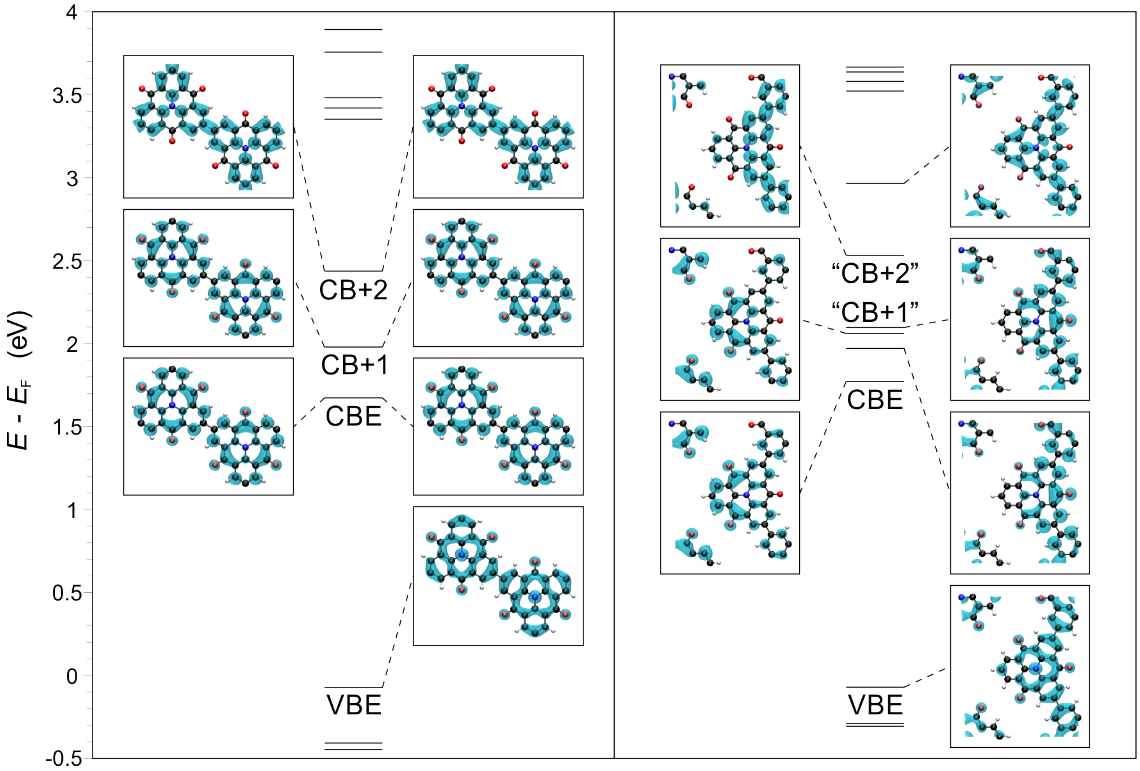

Supplementary Figure 13 | DFT calculated energy levels and partial charge densities in gas phase. Energy levels and partial charge densities (cyan) around the Fermi level for the 2D network (left) and the self-assembled 1D linear chain (right), respectively. The partial charge densities are shown for an isodensity value of 0.005 e/Å^3.

Supplementary Methods

Materials and general methods

Starting materials were purchased reagent grade from Acros and Sigma-Aldrich and used without further purification. O,O',O''-Amino-trisbenzoic acid-trimethylester (**S1**) was synthesized according to literature procedure.[12] Reactions were carried out in flame-dried glassware and under an inert atmosphere of purified N_2 using Schlenk techniques. Thin-layer chromatography (TLC) was performed on the aluminum plates coated with 0.20 mm thickness of Silica Gel 60 F-254 (Macherey-Nagel). Column chromatography was performed on silica gel (230 – 400 mesh). ^{1}H NMR and ^{13}C NMR spectra were recorded on a Bruker Avance 400 spectrometer (400.1 MHz for ^{1}H and 100.6 MHz for ^{13}C) in CD_2Cl_2 at room temperature (r.t.). Chemical shifts (δ) are reported in ppm and were referenced to the residual solvent signal as an internal reference ($CDCl_3$: 7.26 ppm for ^{1}H and 77.16 ppm for ^{13}C; CD_2Cl_2: 5.32 ppm for ^{1}H and 53.8 ppm for ^{13}C). Coupling constants (J) are given in Hz and the apparent resonance multiplicity is reported as s (singlet), d (doublet), t (triplet), q (quartet), and m (multiplet). Infrared spectra (IR) spectra were recorded on a 660-IR (Varian, ATR mode) spectrometer. Characteristic IR absorptions are reported in cm^{-1} and denoted as strong (s), medium (m), and weak (w). Mass spectra were obtained from a MicrOTOF II (Bruker, HR ESI and APPI) and a 9.4T Apex-Qe FTICR (Bruker, (MA)LDI) mass spectrometer. The signal of the molecular ion $[M]^+$ is reported in m/z units.

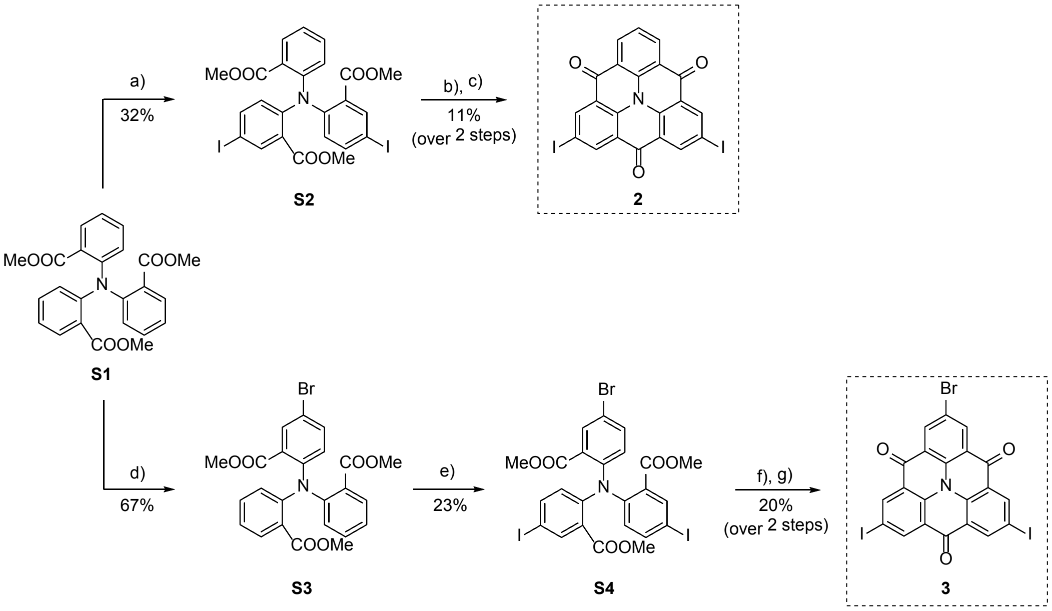

Supplementary Figure 14 | Synthesis of the halogenated CTPA precursors 2 and 3. a) I_2, Ag_2SO_4, EtOH, r.t.; b) KOH, MeOH/H_2O (v/v 4:1), 50 °C; c) $SOCl_2$, $SnCl_4$, DMF, CH_2Cl_2, 40 °C; d) NBS, CH_2Cl_2, r.t.; e) I_2, Ag_2SO_4, EtOH, r.t.; f) KOH, MeOH/H_2O (v/v 4:1), 60 °C; g) $SOCl_2$, $SnCl_4$, DMF, CH_2Cl_2, 40 °C. DMF = *N,N*-dimethylformamide; NBS = *N*-bromosuccinimide.

Experimental procedures

Dimethyl 2,2'-{[2-(methoxycarbonyl)phenyl]imino}bis(5-iodobenzoate) (S2): To a mixture of I_2 (7.26 g, 28.6 mmol) and Ag_2SO_4 (8.90 g, 28.6 mmol) in dry EtOH (350 mL), **S1** (4.00 g, 9.54 mmol) was added. The mixture was stirred under nitrogen atmosphere and exclusion of light for 24 h at r.t.. The solvent was removed under reduced pressure. After addition of CH_2Cl_2 (300 mL), the organic layer was washed with aq. $Na_2S_2O_3$ (10wt%., 100 mL), H_2O (2 × 100 mL), dried ($MgSO_4$), and filtered. The solvent was removed under reduced pressure and the residue was purified by column chromatography (SiO_2, hexanes/EtOAc 3:1) to provide **S2** as a pale yellow solid (2.05 g, 32%), next to its triiodinated counterpart (3.04 g, 40%). R_f = 0.54 (SiO_2, hexanes/EtOAc 3:1); Mp 70–72 °C; ^{1}H NMR (300 MHz, CD_2Cl_2) δ 7.87 (d, J = 4.8 Hz, 2H), 7.66–7.59 (m, 3H), 7.40 (t, J = 7.8 Hz, 1H), 7.17 (t, J = 7.5 Hz, 1H), 7.04 (d, J = 8.4 Hz, 1H), 6.74 (dd, J = 8.7, 2.4 Hz, 2H), 3.41 (s, 3H), 3.37 (s, 3H), 3.35 (s, 3H) ppm; ^{13}C NMR (75 MHz, CD_2Cl_2) δ 167.4, 166.2, 166.1, 146.8, 146.6, 146.2, 141.3, 141.2, 139.82, 139.78, 132.7, 131.4, 129.5, 129.4, 128.5, 128.1, 128.0, 127.3, 124.9, 86.6, 86.5, 52.2, 52.11, 52.08 ppm; IR (ATR) 2944 (w), 1712 (s), 1577 (w), 1470 (m), 1431 (m), 1387 (m), 1282 (s), 1225 (s), 1128 (m), 1080

(s), 960 (m), 817 (m), 758 (s), 712 (m) cm^{-1}; ESI HRMS (toluene) calcd. for $C_{24}H_{19}I_2NO_6$ $[M]^+$ 670.9296, found 670.9298.

2,6-Diiodo-4*H*,8*H*,12*H*-benzo[1,9]quinolizino[3,4,5,6,7-*defg*]acridine-4,8,12-trione (2): **S2** (166 mg, 0.289 mmol) and KOH (243 mg, 4.33 mmol) were dissolved in MeOH (10 mL) and H_2O (3 mL). After stirring at 60°C for 21 h, the solution was diluted with H_2O (10 mL) and acidified (pH 3) with aq. HCl (1 M). The resulting precipitate was filtered off, excessively washed with H_2O, and dried in the oven at 95°C to give the triacid as a pale yellow powder (67.3 mg, 37%) which was used without further purification and characterization for the next step. The triacid (66.8 mg, 0.106 mmol), $SOCl_2$ (379 mg, 0.23 mL, 3.19 mmol), and DMF (20 µL) were stirred in dry CH_2Cl_2 (3 mL) at 40°C for 3 h. After addition of $SnCl_4$ (497 mg, 0.22 mL, 1.91 mmol), the solution was stirred at 40°C for further 18 h. The yellow precipitate was filtered off and stirred in aq. NaOH (1 M, 5 mL) for 30 min. After filtration the residue was excessively washed with H_2O and acetone and dried. The compound **1** was obtained as a yellow solid (18.3 mg, 30%). Mp >400°C. ^1H NMR (300 MHz, $CDCl_3$ + 5vol% trifluoroacetic acid (TFA)) δ 9.38–9.35 (m, 4H), 9.14 (d, J = 7.5 Hz, 2H), 8.01 (t, J = 7.8 Hz, 1H) ppm; ^{13}C NMR: Not available due to low solubility of the compound even after addition of TFA; IR (ATR) 3057 (w), 1650 (s), 1598 (m), 1475 (m), 1435 (s), 1321 (m), 1277 (m), 1018 (m), 917 (w), 793 (s), 769 (m), 707 (m), 669 (m) cm^{-1}; ESI HRMS (CH_3CN, toluene) calcd. for $C_{21}H_7I_2NO_3$ $[M]^+$ 574.8510, found 574.8513.

Methyl 2-{bis[2-(methoxycarbonyl)phenyl]amino}-5-bromobenzoate (S3): To a solution of **S1** (200 mg, 0.477 mmol) in CH_2Cl_2 (40 mL) cooled to 0°C, NBS (85 mg, 0.477 mmol) was added. The mixture was stirred for 30 min at 0°C, subsequently allowed to reach r.t. and stirred for 15 h. After dilution with CH_2Cl_2 (40 mL), the organic layer was washed with H_2O (3 × 30 mL), dried ($MgSO_4$), and filtered. The solvent was removed under reduced pressure and the residue was purified by column chromatography (SiO_2, hexanes/EtOAc 4:1) to provide **S3** as a yellow solid (160 mg, 67%). R_f = 0.55 (SiO_2, hexanes/EtOAc 4:1); Mp 177–179 °C; ^1H NMR (300 MHz, CD_2Cl_2) δ 7.68 (d, J = 2.5 Hz, 1H), 7.61 (m, 2H), 7.46 (dd, J = 8.8, 2.5 Hz, 1H), 7.38 (m, 2H), 7.17 (m, 2H), 7.06 (d, J = 8.2 Hz, 2H), 6.89 (d, J = 8.8, 1H), 3.39 (s, 3H), 3.37 (s, 3H), 3.33 (s, 3H) ppm; ^{13}C NMR (75 MHz, $CDCl_3$) δ 167.4, 166.2, 146.4, 146.3, 146.0, 135.0, 133.5, 132.3, 131.0, 127.9, 127.1, 126.2, 123.4, 115.6, 52.3, 51.3 ppm; IR (ATR) 2947 (w), 1719 (s), 1597 (w), 1437 (s), 1432 (m), 1236 (m), 1281 (s), 1081 (w), 759 (s), 718 (m) cm^{-1}; ESI HRMS (toluene) calcd. for $C_{24}H_{20}BrNO_6$ $[M]^+$ 497.0465, found 497.0468.

Dimethyl 2,2'-{[4-bromo-2-(methoxycarbonyl)phenyl]imino}bis(5-iodobenzoate) (S4): To a mixture of I_2 (153 mg, 0.600 mmol) and Ag_2SO_4 (185 mg, 0.600 mmol) in dry EtOH (6 mL), **S3** (100 mg, 0.200 mmol) was added. The mixture was stirred under nitrogen atmosphere for 15 h at r.t. After dilution with CH_2Cl_2 (10 mL), the organic layer was washed with H_2O (3 × 10 mL), dried ($MgSO_4$), and filtered. The solvent was removed under reduced pressure and the residue was purified by column chromatography (SiO_2, hexanes/EtOAc 4:1) to provide **S4** as a yellow solid (35 mg, 23%). R_f = 0.85 (SiO_2, hexanes/EtOAc 4:1); Mp 171–173°C; ^1H NMR (300 MHz, $CDCl_3$) δ 7.89 (d, J = 2.1 Hz, 2H), 7.72 (d, J = 2.4 Hz, 1H), 7.64 (dd, J = 8.6, 2.2 Hz, 2H), 7.47 (dd, J = 8.7, 2.5 Hz, 1H), 6.91 (d, J = 8.7 Hz, 1H), 6.76 (dd, J = 8.6, 1.5 Hz, 2H), 3.42 (s, 3H), 3.41 (s, 6H) ppm; ^{13}C NMR (75 MHz, $CDCl_3$) δ 165.7, 165.6, 145.84, 145.81, 145.0, 141.1, 139.5, 135.4, 133.7, 129.9, 127.6, 116.9, 87.0, 51.7, 51.3 ppm; IR (ATR) 2947 (w), 1722 (s), 1471 (s), 14339 (s), 1389 (m), 1282 (s), 1227 (s), 1146 (m), 1084 (s), 964 (m), 907 (m), 820 (m), 729 (s) cm^{-1}; ESI HRMS (toluene) calcd. for $C_{24}H_{18}BrI_2NO_6$ $[M]^+$ 748.8409, found 748.8401.

2-Bromo-6,10-diiodo-4*H*,8*H*,12*H*-benzo[1,9]quinolizino[3,4,5,6,7-*defg*]acridine-4,8,12-trione (3): **S4** (48 mg, 0.064 mmol) and KOH (52 mg, 0.927 mmol) were dissolved in MeOH (8 mL) and H_2O (2 mL). After stirring at 60°C for 20 h, the solution was diluted with H_2O (10 mL) and acidified (pH 3) with aq. HCl (1 M). The resulting precipitate was filtered off, excessively washed with H_2O, and dried in the oven at 95°C to give the triacid as a colorless powder (13 mg, 29%) which was used without further purification and characterization for the next step. The triacid (13 mg, 0.018 mmol), $SOCl_2$ (65 mg, 40 µL, 0.55 mmol), and DMF (10 µL) were stirred in dry CH_2Cl_2 (0.5 mL) at 40°C for 3 h. After addition of $SnCl_4$ (84 mg, 38 µL, 0.33 mmol), the solution was stirred at 40°C for further 22 h. The yellow precipitate was filtered off and stirred in aq. NaOH (1 M, 20 mL) for 30 min. After filtration, the residue was excessively washed with H_2O and acetone and dried. The compound **2** was obtained as a yellow solid (8.0 mg, 68%). Mp >400°C; ^1H and ^{13}C NMR: Not available due to low solubility of the compound even after addition of TFA; IR (ATR) 3061 (w), 1654 (s), 1594 (s), 1440 (s), 1321 (s), 1279 (s), 1099 (w), 799 (m), 718 (m) cm^{-1}. ESI HRMS (CH_3CN, toluene) calcd. for $C_{21}H_6BrI_2NO_3$ $[M]^+$ 652.7615, found 652.7612.

Supplementary References

1. Bieri M*, et al.* Surface-supported 2D heterotriangulene polymers. *Chem. Comm.* **47**, 10239-10241 (2011).
2. Temirov R, Soubatch S, Neucheva O, Lassise AC, Tautz FS. A novel method achieving ultra-high geometrical resolution in scanning tunnelling microscopy. *New J. Phys.* **10**, 053012 (2008).
3. Weiss C, Wagner C, Kleimann C, Rohlfing M, Tautz FS, Temirov R. Imaging Pauli repulsion in scanning tunneling microscopy. *Phys. Rev. Lett.* **105**, 086103 (2010).
4. Gross L, Mohn F, Moll N, Liljeroth P, Meyer G. The chemical structure of a molecule resolved by atomic force microscopy. *Science* **325**, 1110-1114 (2009).
5. Chiang C-l, Xu C, Han Z, Ho W. Real-space imaging of molecular structure and chemical bonding by single-molecule inelastic tunneling probe. *Science* **344**, 885-888 (2014).
6. Hapala P*, et al.* Mapping the electrostatic force field of single molecules from high-resolution scanning probe images. *Nat. Commun.* **7**, 11560 (2016).
7. Hapala P, Kichin G, Wagner C, Tautz FS, Temirov R, Jelínek P. Mechanism of high-resolution stm/afm imaging with functionalized tips. *Phys. Rev. B* **90**, 085421 (2014).
8. Lobo-Checa J*, et al.* Band formation from coupled quantum dots formed by a nanoporous network on a copper surface. *Science* **325**, 300-303 (2009).
9. Müller K, Enache M, Stöhr M. Confinement properties of 2D porous molecular networks on metal surfaces. *J. Phys.: Condens. Matter* **28**, 153003 (2016).
10. Vázquez H, Dappe YJ, Ortega J, Flores F. Energy level alignment at metal/organic semiconductor interfaces: "Pillow" effect, induced density of interface states, and charge neutrality level. *J. Chem. Phys.* **126**, 144703 (2007).
11. Gebhardt J, Viñes F, Görling A. Influence of the surface dipole layer and Pauli repulsion on band energies and doping in graphene adsorbed on metal surfaces. *Phys. Rev. B* **86**, 195431 (2012).
12. Fang Z, Teo T-L, Cai L, Lai Y-H, Samoc A, Samoc M. Bridged triphenylamine-based dendrimers: Tuning enhanced two-photon absorption performance with locked molecular planarity. *Org. Lett.* **11**, 1-4 (2009).
